# Correlation analysis of vaginal microecology and different types of human papillomavirus infection: a study conducted at a hospital in northwest China

**DOI:** 10.3389/fmed.2023.1138507

**Published:** 2023-06-01

**Authors:** Fang Feng, Yue-min Hou, Yan Zhang, Lu-yuan Wang, Pei-pei Li, Ying Guo, Rui-fang An

**Affiliations:** ^1^Department of Gynecology and Obstetrics, The First Affiliated Hospital of Xi'an Jiaotong University, Xi'an, Shaanxi, China; ^2^Department of Ophthalmology, The First Affiliated Hospital of Xi'an Jiaotong University, Xi'an, Shaanxi, China

**Keywords:** vaginal microecology, human papillomavirus (HPV), mixed vaginitis, bacterial vaginosis, vulvovaginal candidiasis

## Abstract

**Background:**

Vaginal microecology has a definite influence on human papillomavirus (HPV) infection and clearance, but the specific correlation is still controversial. This research aimed to investigate the differences in the vaginal microenvironment of different types of HPV infection and also provide data supporting clinical diagnosis and treatment.

**Methods:**

According to strict inclusion and exclusion criteria, the case data of 2,358 female patients who underwent vaginal microecology and HPV-DNA tests at the same time in the Department of Obstetrics and Gynecology of the First Affiliated Hospital of Xi'an Jiaotong University from May 2021 to March 2022 were retrospectively analyzed. The population was divided into two groups: an HPV-positive group and an HPV-negative group. HPV-positive patients were further classified into HPV16/18-positive group and HPV other subtypes positive group. The vaginal microecology of HPV-infected patients was analyzed using the chi-square test, Fisher's exact test, and logistic regression.

**Results:**

Among the 2,358 female patients, the HPV infection rate was 20.27% (478/2,358), of which the HPV16/18 infection rate was 25.73% (123/478), and the HPV other subtypes infection rate was 74.27% (355/478). The difference in HPV infection rates between the age groups was statistically significant (*P* < 0.01). The prevalence of mixed vaginitis was 14.37% (339/2,358), with bacterial vaginosis (BV) paired with aerobic vaginitis (AV) accounting for the majority (66.37%). The difference in HPV infection rates among mixed vaginitis was not statistically significant (*P* > 0.05). The prevalence of single vaginitis was 24.22% (571/2,358), with the most frequent being vulvovaginal *Candidiasis* (VVC; 47.29%, 270/571), and there was a significant difference in HPV infection rates among single vaginitis (*P* < 0.001). Patients with BV had a higher risk of being positive for HPV16/18 (OR: 1.815, 95% CI: 1.050–3.139) and other subtypes (OR: 1.830, 95% CI: 1.254–2.669). Patients with *Trichomoniasis* were at higher odds of other HPV subtype infections (OR: 1.857, 95% CI: 1.004–3.437). On the contrary, patients with VVC had lower odds of becoming infected with other HPV subtypes (OR: 0.562, 95% CI: 0.380–0.831).

**Conclusion:**

There were disparities in HPV infection among different age groups; therefore, we should pay attention to the prevention and treatment of susceptible individuals. BV and *Trichomoniasis* are linked to HPV infection; hence, restoring the balance of vaginal microecology could assist in the prevention of HPV infection. As a protective factor for other HPV subtype infections, VVC may provide new insights into the development of immunotherapeutic therapies.

## Introduction

In 2020, cervical cancer had the fourth highest incidence and mortality rate among female patients, with an estimated 604,000 new cases and 342,000 deaths worldwide (1). High-risk human papillomavirus (HR-HPV) persistent infection, especially HPV16/18 infection, is considered to be a key factor in the occurrence of cervical cancer. Although most HPV infectious cases can be cleared in the first 2 years (2), the factors that promote HPV persistence and trigger carcinogenic pathways are not fully understood. In recent years, vaginal microecology has become a research hotspot for various gynecological diseases, and a variety of vaginal microbiota and related inflammation has been found to be potential drivers of HPV infection and disease severity (3). Previous studies have revealed that bacterial vaginosis (BV) and decreased *Lactobacilli* are associated with an increased risk of HPV infection (4, 5), while the relationships between *Trichomoniasis*, vulvovaginal *Candidiasis* (VVC), and other vaginal microdysbiosis and HPV infection are controversial (5–7). After collecting the results of vaginal microecology examinations, HPV-DNA tests, and related clinical data, this study aimed to clarify the correlation between vaginal microecology and HPV infection, so as to provide solid guidance for reducing the odds of HPV infection and preventing cervical cancer.

## Materials and methods

### Population screening

From May 2021 to March 2022, a total of 2,358 patients from the physical examination center, assisted reproductive technology center, and gynecology clinics were enrolled in this study. They underwent vaginal microecology and HPV-DNA tests during the same period at the First Affiliated Hospital of Xi'an Jiaotong University, Shaanxi Province, China. The median [interquartile range (IQR)] age of participants was 35 (28–42) years. The inclusion criteria are as follows: participants who (1) had a sexual history; (2) had no gynecological examination or vaginal ultrasound within 24 h; (3) are at a non-menstrual stage; and (4) had accepted HPV-DNA tests in our hospital. The exclusion criteria are as follows: participants who (1) are pregnant or lactating; (2) had sexual intercourse or used a drug in the vagina within the last 3 days; (3) used antibiotics within 1 month; (4) had accompanying autoimmune diseases or being under immunosuppressive treatment; (5) are complicated with serious medical or surgical diseases; (6) had the history of cervical lesion or HPV infection treatment, including medical and surgical treatments; and (7) had the history of HPV vaccination. The flow chart of the screening process is shown in the Supplementary Figure 1. This study was approved by the Medical Ethics Committee of the First Affiliated Hospital of Xi'an Jiaotong University, Xi'an, China.

### Detection methods

Two sterile cotton swabs were used to collect secretions from the upper 1/3 of the vagina; the swabs were placed in test tubes for immediate testing. A special brush for HPV-DNA testing was placed into the cervical canal and rotated 3–5 times; the brush was removed and placed into a specimen tube containing cell preservation solution, then the tube cap was closed and the samples were sent for testing.

### Testing and diagnosis

One of the sample cotton swabs was used to make two slide preparations, one to microscopically observe *Trichomoniasis* and perform a Donders score using the physiological saline wet sheet method; and the other was used to observe the density and diversity of flora, pseudohyphae, spores, Nugent score, and *Lactobacilli* grading, as viewed microscopically under oil after drying, fixing, and gram staining. We used the Vaginitis Combined Test Kit provided by Jiangsu Shuoshi Biotechnology Co., Ltd., to detect vaginal microbial function and inflammatory response indicators using the specimens on the second set of cotton swabs, including pH value, leukocyte esterase (LE), neuraminidase (SNA), β-glucuronidase (GUS), and β-N-acetylglucosaminidase (NAG). Evaluation and diagnosis were based on the Vaginal Microecology Evaluation System v.2016 (8) as follows: (1) Normal vaginal microecology: density and diversity grades were II–III, the dominant bacteria were *Lactobacilli*, no pathogenic microorganisms were detected, vaginal pH value was 3.8–4.5, *Lactobacilli* grade was I–IIa, and inflammatory response indicators were negative; (2) BV: Nugent score ≥7 points; (3) VVC: fungal spores or pseudohyphae could be found microscopically under oil; (4) aerobic vaginitis (AV): according to the clinical manifestations and microscopic Donders score ≥3 points; (5) *Trichomoniasis*: a large number of white blood cells and active *Trichomoniasis* under the microscope; (6) Mixed vaginitis: two or more types of vaginitis existing at the same time; and (7) Microbial function and inflammatory response indicators could be determined according to the test kit.

DNA from cervical cells was extracted for hybridization and color development. A total of 15 high-risk HPV (16, 18, 31, 33, 35, 39, 45, 51, 52, 53, 56, 58, 59, 66, and 68) and 6 low-risk HPV (6, 11, 26, 73, 81, and 82) types can be detected by HPV-DNA detection kit provided by Jiangsu Shuoshi Biotechnology Co., Ltd. The evaluation and diagnosis results were classified into (1) “HPV-positive”: one or more of the above-mentioned HPV subtypes were detected and divided into “HPV16/18-positive group” and “HPV other subtype positive group”; and (2) “HPV-negative”: none of the above HPV subtypes were detected.

### Statistical methods

SPSS 25.0 and Origin 2019 software were used for statistical analysis and graphing, respectively. Quantitative data were expressed as median (IQR) and qualitative data were described by absolute and relative indices. The chi-square test, Fisher's exact probability test, or univariate logistic regression analysis were used for the comparison of factors between the groups, the multivariate logistic regression analysis was carried out with significant results, and the OR value and 95% CI were calculated. Statistical tests were two-sided; *P* < 0.05 was considered statistically significant.

## Results

### HPV infection at different age ranges

**Figure 1:**
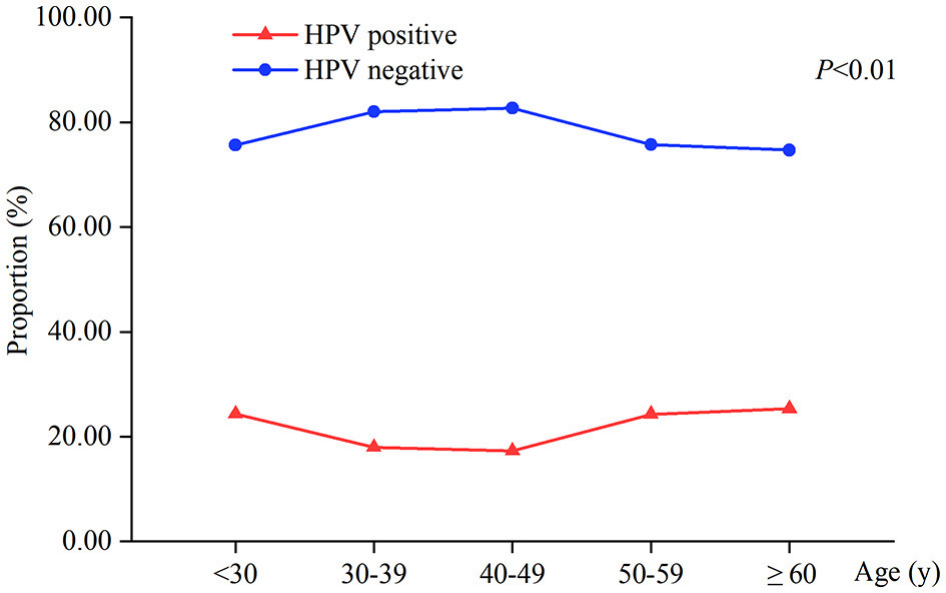
HPV infection at different age ranges in 2,358 patients.

The total HPV infection rate of 2,358 patients was 20.27% (478/2,358). There was a statistically significant difference in HPV infection between the different age groups (*P* < 0.01). The HPV-positive rates of women aged <30, 50–59, and ≥60 years were 24.34%, 24.30%, and 25.33%, respectively, higher than 17.29%−17.98% of women aged 30–49 years (Table 1, Figure 1).

**Table 1:** HPV infection at different age ranges in 2,358 patients.

**Age (y)**	**Total *n***	**HPV positive**	**HPV negative**	**χ^2^**	***P*-value**
		***n*** **(%)**	***n*** **(%)**		
< 30	571	139 (24.34)	432 (75.66)	15.518	**0.004**
30–39	929	167 (17.98)	762 (82.02)		
40–49	532	92 (17.29)	440 (82.71)		
50–59	251	61 (24.30)	190 (75.70)		
≥60	75	19 (25.33)	56 (74.67)		
Total	2,358	478 (20.27)	1,880 (79.73)		

Bold value: P < 0.05.

### HPV-positive types at different age distributions

**Figure 2:**
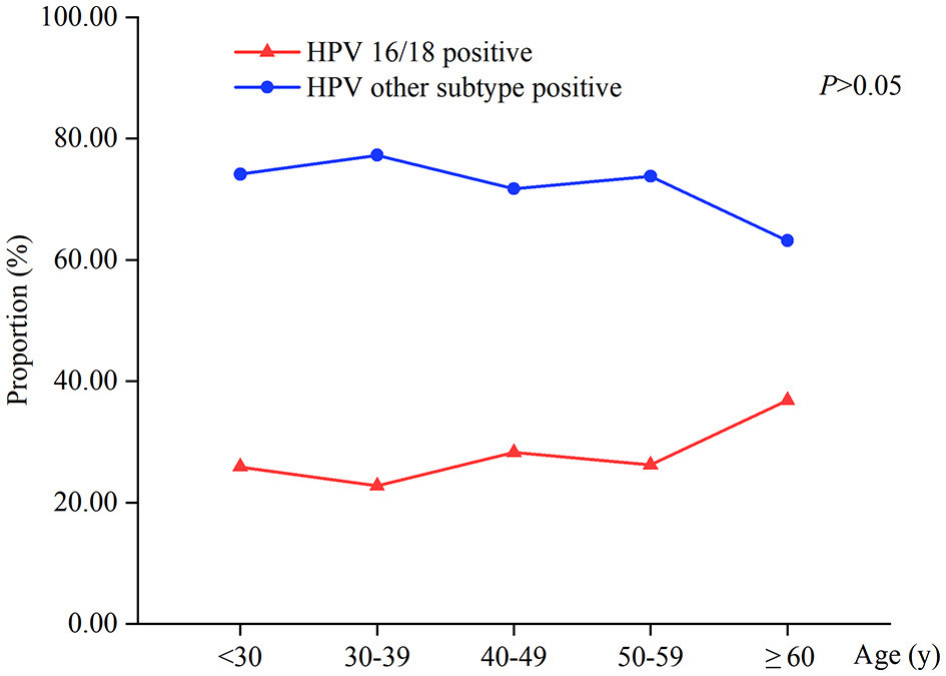
HPV-positive types at different age distributions in 478 patients.

The incidence of HPV16/18-positive was 25.73% (123/478), while the rate of HPV other subtypes was 74.27% (355/478). The difference in HPV16/18-positive rate between different age groups was not statistically significant (*P* > 0.05). HPV16/18 infection was most common in women aged ≥60 years, with a positive rate of 36.84%; the lowest positive rate was in the 30–39 years group (22.75%). HPV other subtypes infection was more common in women aged 30–39 years, and the positive rate was 77.25%. On average, the infection rates of HPV other subtypes in different age groups were three times higher than that of HPV16/18 (Table 2, Figure 2).

**Table 2:** HPV-positive types at different age distributions in 478 patients.

**Age (year)**	**HPV positive**	**HPV16/18**	**HPV other subtypes**	**χ^2^**	***P*-value**
	***n***	***n*** **(%)**	***n*** **(%)**		
< 30	139	36 (25.90)	103 (74.10)	2.487	0.652
30–39	167	38 (22.75)	129 (77.25)		
40–49	92	26 (28.26)	66 (71.74)		
50–59	61	16 (26.23)	45 (73.77)		
≥60	19	7 (36.84)	12 (63.16)		
Total	478	123 (25.73)	355 (74.27)		

### HPV infection in patients with different types of vaginitis

Among the 2,358 patients, 471 cases had normal vaginal microecology; there were 1,887 cases of abnormalities, including 339 cases of mixed vaginitis, 571 cases of single vaginitis, and 977 cases of vaginal microdysbiosis of an unknown pathogen. BV + AV accounted for the largest number (66.37%, 225/339) in patients with mixed vaginitis. In mixed vaginitis, the difference in HPV infection rates was not statistically significant (*P* > 0.05). VVC was the most common single vaginitis (47.29%, 270/571), and there was a significant difference in HPV infection rates among single vaginitis (*P* < 0.001; Table 3).

**Table 3:** HPV infection in patients with different types of vaginitis.

**Types of vaginitis**	**Total, *n***	**HPV positive**	**HPV negative**	χ^2*^	***P*-value**
		***n*** **(%)**	***n*** **(%)**		
Mixed vaginitis	339	103 (30.38)	236 (69.62)	5.845	0.566
BV + AV	225	72 (32.00)	153 (68.00)		
VVC + BV + AV	35	7 (20.00)	28 (80.00)		
TV + BV + AV	27	10 (37.04)	17 (62.96)		
TV + AV	22	5 (22.73)	17 (77.27)		
VVC + BV	16	5 (31.25)	11 (68.75)		
VVC + AV	12	3 (25.00)	9 (75.00)		
TV + BV	1	1 (100.00)	0 (0.00)		
TV + VVC + AV	1	0 (0.00)	1 (100.00)		
Single vaginitis	571	105 (18.39)	466 (81.61)	21.483	**< 0.001**
VVC	270	34 (12.59)	236 (87.41)		
AV	148	25 (16.89)	123 (83.11)		
BV	144	42 (29.17)	102 (70.83)		
TV	9	4 (44.44)	5 (55.56)		

BV, bacterial vaginosis; AV, aerobic vaginitis; VVC, vulvovaginal candidiasis; TV, *Trichomoniasis*.

^*^The values were calculated by chi-square test or Fisher exact probability test.

Bold value: P < 0.05.

### Different types of vaginitis in HPV-positive patients

HPV-positive patients with mixed vaginitis and single vaginitis accounted for 21.55% (103/478) and 21.97% (105/478), respectively. BV+AV was the most common mixed vaginitis in HPV-positive patients (69.90%, 72/103), and BV was the most common single vaginitis (40.00%, 42/105). There was no significant difference in the HPV16/18 infection rate among different types of vaginitis either for mixed vaginitis or single vaginitis (*P* > 0.05; Table 4).

**Table 4:** Different types of vaginitis in HPV-positive patients.

**Types of vaginitis**	**HPV positive**	**HPV16/18**	**HPV other subtypes**	χ^2*^	***P*-value**
	***n***	***n*** **(%)**	***n*** **(%)**		
Mixed vaginitis	103	27 (26.21)	76 (73.79)	1.955	0.975
BV + AV	72	19 (26.39)	53 (73.61)		
TV + BV + AV	10	2 (20.00)	8 (80.00)		
VVC + BV + AV	7	2 (28.57)	5 (71.43)		
TV + AV	5	1 (20.00)	4 (80.00)		
VVC + BV	5	2 (40.00)	3 (60.00)		
VVC + AV	3	1 (33.33)	2 (66.67)		
TV + BV	1	0 (0.00)	1 (100.00)		
Single vaginitis	105	31 (29.52)	74 (70.48)	3.301	0.348
BV	42	12 (28.57)	30 (71.43)		
VVC	34	13 (38.24)	21 (61.76)		
AV	25	6 (24.00)	19 (76.00)		
TV	4	0 (0.00)	4 (100.00)		

BV, bacterial vaginosis; AV, aerobic vaginitis; VVC, vulvovaginal candidiasis; TV, *Trichomoniasis*.

^*^The values were calculated by chi-square test or Fisher exact probability test.

### Determinants of HPV16/18 positive

Table 5 shows distributions of patient characteristics according to the status HPV16/18 positive, using univariable and multivariable logistic regression. The differences in the incidence of BV and abnormal *Lactobacilli* grade were statistically significant between HPV16/18-positive and HPV-negative groups (*P* < 0.05). On the multivariable logistic regression, women with BV (OR: 1.815, 95% CI: 1.050–3.139) had higher odds of being HPV16/18 positive.

**Table 5:** The vaginal microecological status between HPV16/18-positive and HPV-negative groups.

**Vaginal microecological status**	**Total, *n***	**HPV16/18 positive**	**HPV negative**	**Univariate analysis**		**Multivariate analysis**	
		***n*** **(%)**	***n*** **(%)**	***P*** **-value**	**OR (95% CI)**	***P*** **-value**	**OR (95% CI)**
Density	N:1,869	116 (94.31)	1,753 (93.24)	0.648	0.833 (0.380–1.824)		
	A:134	7 (5.69)	127 (6.76)				
Diversity	N:1,870	119 (96.75)	1,751 (93.14)	0.129	0.456 (0.166–1.256)		
	A:133	4 (3.25)	129 (6.86)				
TV	43	3 (2.44)	40 (2.13)	0.818	1.150 (0.351–3.771)		
VVC	303	18 (14.63)	285 (15.16)	0.875	0.959 (0.573–1.607)		
BV	348	37 (30.08)	311 (16.54)	**< 0.001**	2.171 (1.449–3.251)	**0.033**	1.815 (1.050–3.139)
AV	379	31 (25.20)	348 (18.51)	0.068	1.483 (0.971–2.265)		
LE	N:1,059	63 (51.22)	996 (52.98)	0.705	1.073 (0.745–1.546)		
	A:944	60 (48.78)	884 (47.02)				
SNA	N:1,869	111 (90.24)	1,758 (93.51)	0.163	1.558 (0.835–2.905)		
	A:134	12 (9.76)	122 (6.49)				
NAG	N:1,902	115 (93.50)	1,787 (95.05)	0.446	1.337 (0.634–2.819)		
	A:101	8 (6.50)	93 (4.95)				
pH	N:839	50 (40.65)	789 (41.97)	0.774	1.056 (0.728–1.531)	0.178	0.739 (0.476–1.147)
	A:1,164	73 (59.35)	1,091 (58.03)				
*Lactobacilli* grade	N:1,297	64 (52.03)	1,233 (65.59)	**0.003**	1.757 (1.218–2.534)	0.167	1.472 (0.851–2.544)
	A:706	59 (47.97)	647 (34.41)				
Total	2,003	123	1,880				

TV, *Trichomoniasis*; VVC, vulvovaginal candidiasis; BV, bacterial vaginosis; AV, aerobic vaginitis; LE, leukocyte esterase; SNA, neuraminidase; NAG, β-N-acetylglucosaminidase; GUS, β-glucuronidase, all the GUS in this study were negative; OR, odds ratio; CI, confidence interval; N, normal; A, abnormal.

Bold values: P < 0.05.

### Determinants of HPV other subtypes positive

Table 6 shows distributions of patient characteristics according to HPV other subtypes positive by univariable and multivariable logistic regression. There were significant differences in *Trichomoniasis*, VVC, BV, AV, abnormal SNA, and *Lactobacilli* grade between HPV other subtypes positive and HPV-negative groups (*P* < 0.05). Multivariate logistic regression showed that patients with *Trichomoniasis* (OR: 1.857, 95% CI: 1.004–3.437) or BV (OR: 1.830, 95% CI: 1.254–2.669) had higher odds of being positive for HPV other subtypes; on the contrary, patients with VVC (OR: 0.562, 95% CI: 0.380–0.831) had lower odds of becoming infected with HPV other subtypes.

**Table 6:** The vaginal microecological status between HPV other subtypes positive and HPV-negative groups.

**Vaginal microecological status**	**Total, *n***	**HPV other subtypes positive**	**HPV negative**	**Univariate analysis**		**Multivariate analysis**	
		***n*** **(%)**	***n*** **(%)**	***P*** **-value**	**OR (95% CI)**	***P*** **-value**	**OR (95% CI)**
Density	N:2,086	333 (93.80)	1,753 (93.24)	0.699	0.912 (0.571–1.455)		
	A:149	22 (6.20)	127 (6.76)				
Diversity	N:2,088	337 (94.93)	1,751 (93.14)	0.214	0.725 (0.437–1.203)		
	A:147	18 (5.07)	129 (6.86)				
TV	57	17 (4.79)	40 (2.13)	**0.005**	2.314 (1.296–4.129)	**0.049**	1.857 (1.004–3.437)
VVC	316	31 (8.73)	285 (15.16)	**0.002**	0.535 (0.363–0.790)	**0.004**	0.562 (0.380–0.831)
BV	411	100 (28.17)	311 (16.54)	**< 0.001**	1.978 (1.523–2.569)	**0.002**	1.830 (1.254–2.669)
AV	439	91 (25.63)	348 (18.51)	**0.002**	1.517 (1.164–1.978)	0.769	0.948 (0.666–1.351)
LE	N:1,181	185 (52.11)	996 (52.98)	0.764	1.035 (0.825–1.299)		
	A:1,054	170 (47.89)	884 (47.02)				
SNA	N:2,079	321 (90.42)	1,758 (93.51)	**0.037**	1.526 (1.025–2.273)	0.775	0.937 (0.598–1.466)
	A:156	34 (9.58)	122 (6.49)				
NAG	N:2,127	340 (95.77)	1,787 (95.05)	0.561	0.848 (0.485–1.480)		
	A:108	15 (4.23)	93 (4.95)				
pH	N:925	136 (38.31)	789 (41.97)	0.200	1.165 (0.923–1.470)	0.389	0.886 (0.673–1.167)
	A:1,310	219 (61.69)	1,091 (58.03)				
*Lactobacilli* grade	N:1,427	194 (54.65)	1,233 (65.59)	**< 0.001**	1.582 (1.257–1.990)	0.368	1.180 (0.823–1.690)
	A:808	161 (45.35)	647 (34.41)				
Total	2,235	355	1,880				

TV, *Trichomoniasis*; VVC, vulvovaginal candidiasis; BV, bacterial vaginosis; AV, aerobic vaginitis; LE, leukocyte esterase; SNA, neuraminidase; NAG, β-N-acetylglucosaminidase; GUS, β-glucuronidase, all the GUS in this study were negative; OR, odds ratio; CI, confidence interval; N, normal; A, abnormal.

Bold values, P < 0.05.

## Discussion

In a 2021 study of 36 types of cancer in 186 countries, researchers found that cervical cancer was the most common cancer in 23 countries and was the leading cause of cancer death in 36 countries (1); it has remained a globally important public health issue. At present, the significance of HR-HPV infection, especially HPV16/18 persistent infection, for cervical cancer has been recognized by most clinicians. Our study showed that the HPV infection rate was 20.27%, a decrease from 22.97% in the same region previously (9), which may be related to higher education, widespread HPV vaccination, or an increase in average socioeconomic level (10, 11). HPV infection rate was higher in women aged <30 and ≥50 years; this was consistent with the previously reported double-peak distribution of HPV infection rates, which was considered to be related to the frequent sexual life, smoking, and oral hormonal contraceptive use in young women (11, 12), as well as the changes in sexual behavior, low autoimmunity, and the reactivation of menopausal latent HPV in older women (13, 14).

In this study, HPV16/18 infection rate was 25.73%, and it was more common in women ≥60 years, which was similar to findings in a previous study (9). Zhang et al. (15) found that the incidence of cervical cancer for women aged >60 years was 18.52%, while it was 0.99% for those aged ≤30 years, suggesting the existence of substantial cervical cancer risk for older women. We compared the vaginal microecology of patients with HPV16 single-type infection and those with HPV16 combined multiple-type infection in our research. However, we found no significant difference between them. Similarly, there was no statistically significant difference in the vaginal microecological status between the HPV18 single-type infection group and the HPV18 combined multiple-type infection group. We also found an interesting feature that the infection rates of HPV other subtypes in different age groups were, on average, three times higher than that of HPV16/18. This may be caused by differing invasiveness in various HPV types (16) and some cross-protection between HPV 16, 18, 6, 11, 31, and 45 (17). The reasons for this unique characteristic are not clear and should be investigated further. Although the HPV infection rate in young women is high, most infections are transient (2). From the specific age curve of cervical cancer, the incidence increased from the age of 25 years (18). Through a balanced decision analysis of the pros and cons, cervical cancer screening is strongly recommended by the American Cancer Society (ACS) from age 25 years using the primary HPV test (19).

Mixed vaginitis is the simultaneous presence of at least two types of vaginitis, contributing to an abnormal vaginal milieu. Nevertheless, the signs and symptoms of mixed vaginitis are often atypical, and treatment is complicated in contrast to single-type vaginitis (20). Owing to differences in race, region, detection technology, and methods, the incidence of mixed vaginitis is reported to vary greatly, from 4.44 to 35.06% (20, 21); meanwhile, the result in our study was 14.37%, of which BV combined with AV was the most common type (66.37%). Currently, little is known about the vaginal pathophysiology of mixed vaginitis. Studies have found that there were synergistic effects between different pathogens, allowing them to form multi-strain biofilms and to further lead to refractory infections and recurrences (21, 22). Bacteria and/or fungi can coexist within a host and influence each other via synergistic or antagonistic interactions (23). Furthermore, HPV infection can change host immunity or mucosal metabolism so that the genital tract microenvironment changes, making it easier for other infectious diseases to take hold (16). The latest consensus on mixed vaginitis also mentions that the issue of simultaneous infection of the cervix and vagina needs to be addressed while paying attention to the mixed infection of various vaginal inflammations, including *Chlamydia trachomatis, Neisseria gonorrhoeae, Mycoplasma*, and HPV (24).

The novelty of this study is that it investigates the correlation between mixed vaginitis and HPV infection, an area that has limited research. Research considered that mixed vaginitis was related to HPV infection by having higher species diversity and significantly fewer *Lactobacilli* (25). However, our study did not find any differences in mixed vaginitis among the different groups of HPV. Further basic research is needed to verify and clarify this correlation in the future.

Since AV was first proposed in 2002 (26), the research has gradually increased. Some researchers believe that AV is not related to HPV infection (27), while others consider that the infection status of AV patients is related to HR-HPV susceptibility of cervical epithelial cells (28). Meanwhile, studies also found no statistical relationship between moderate-to-severe AV and HR-HPV infection, but it may be involved in the progression of HR-HPV-infected women to cervical intraepithelial neoplasia and cancer (29); in turn, this was related to the inhibition of innate immune response due to their chronic inflammatory features. The AV-associated interleukin (IL) profile was consistent with that found in patients with HR-HPV infection progressing to cancer. Our study found that, compared with the HPV negative group, the infection rate of AV in the HPV other subtypes positive group was higher, but it was not an independent risk factor for the other subtypes positive group. Because of the high proportion (68.51%) of AV patients combined with the other vaginitis in this study, and mainly combined with BV, we suggest that different pathogens influenced each other, which may have affected the interpretation of their correlation. Increasingly, studies have shown a higher incidence of AV-related mixed vaginitis (30, 31), and the relationship between AV itself and HPV infection requires further investigation.

Previous studies have shown that the prevalence of HR-HPV is higher in patients with BV (32). Furthermore, a prospective study has revealed that BV is associated with persistent HPV infection, and the HPV clearance rate is reduced in women with BV (33), which is consistent with our results. It may be that an endogenous mixed infection caused by decreased *Lactobacilli* in the vagina, increased *Gardnerella*, and other anaerobic bacteria in BV increases the content of mucinolytic enzymes in the vagina and disrupts the local mucosal barrier, promoting viral adhesion, invasion, and even integration into the host genome, thereby increasing susceptibility to HPV infection. At the same time, anaerobic bacteria can produce ammonia and carcinogenic nitrites, resulting in abnormal lesions of the cervical epithelium (34).

The main type of single vaginitis varied widely owing to region, ethnicity, and population origin (5, 29). VVC was the most common vaginitis in our study. There is still controversy about the relationship between VVC and HPV infection; while VVC has been reported to not be related to HPV infection (5), another study found that VVC had a high incidence in the HPV infection group (34), which may be related to the inflammation and the increased mucosal permeability of the invasive enzymes caused by infection. However, a meta-analysis also found that VVC may be a protective factor against HPV infection (35); similarly, our study showed that VVC could reduce the risk of infection in HPV other subtypes. *Candida albicans* exhibits colonization; it is usually a harmless member of the native microbiota (23), and can promote T-cell proliferation and enhance immune response (35), which gives protection against HPV other subtypes. Moreover, researchers found that a skin test agent extracted from *C. albicans* and used as an adjuvant for a new HPV therapeutic vaccine can induce the immune response of HPV-specific CD8+ T cells and Th1 CD4^+^ T cells and can also induce humoral immunity (36). This indicates that *C. albicans* is a potential immunotherapeutic agent and could be employed in the development of new vaccines or the treatment of HPV infection and even other diseases.

*Trichomoniasis* is also a common sexually transmitted disease, associated with multiple health consequences in men and women. Our study found that *Trichomoniasis* can increase the risk of infection of HPV other subtypes, consistent with previous studies (37). This may be owing to the release of lytic enzymes by *Trichomoniasis*, which reduces the protective mucus layer of the vagina, leading to the development of epithelial cell microlesions, increasing the erosiveness of other subtypes of HPV, and allowing their DNA to integrate into host cells. This inflammatory process can also destroy the basal layer of the cervical epithelium, thus promoting the continued presence of HPV in cervical–vaginal epithelial tissue (38).

It has been shown that HPV infection is associated with an increase in floral diversity and a decrease in the number of *Lactobacilli* in the vaginal microecological environment (25). Our study found that the diversity of microbiota was not related to HPV infection; however, *Lactobacilli* grade and SNA were associated with infection of HPV other subtypes. We believe that the presence of normal *Lactobacilli* plays an important role in maintaining the acidic environment of the vagina and preventing the invasion of pathogenic microorganisms. Nevertheless, *Lactobacilli* still cannot produce sufficient protective effect against HPV16/18. As the main pathogen of BV, the detection rate of *Gardnerella* was significantly increased in HR-HPV-positive women (32); meanwhile, *Gardnerella* secretes SNA while elevated SNA concentration was associated with increased risk for cervical lesion and HR-HPV persistent infection (39, 40). A case–control study found that there was an interaction effect with abnormities of GUS and SNA on CIN2/3 in the HPV16-negative group, while there was an interaction effect with abnormities of GUS and SNA on CIN in the HPV16-positive group (41). Studies have also shown that the difference in positive rates of GUS between the HPV-positive and HPV-negative groups was not statistically significant (42). Furthermore, few studies have addressed the correlation between NAG and HPV infections. Therefore, further investigations are needed to determine the correlation among NAG, GUS, and HPV infections.

This is a cross-sectional study. To clarify the correlation between vaginal microbiota and different types of HPV infection, it is necessary to expand the sample size in multiple centers and design prospective studies, which is also a common limitation of this study and most other related studies. In addition, we all know that the vaginal microbiome is an intricate and dynamic microecosystem that constantly undergoes fluctuations during the female menstrual cycle and the woman's entire life (43, 44), which may influence the conclusion of the study. It is another limitation of this study.

In conclusion, our study from a hospital in northwest China found differences in HPV infection rates within different age ranges, which offers insights into the prevention of infection at different ages. Vaginal infectious diseases play an important role in HPV infection, and patients with BV or *Trichomoniasis* should be alerted to this interaction. Meanwhile, exploring the mechanism of action of VVC may assist in the development of new vaccines or immunotherapeutic agents.

## Data availability statement

The raw data supporting the conclusions of this article will be made available by the authors, without undue reservation.

## Ethics statement

The studies involving human participants were reviewed and approved by Medical Ethics Committee of the First Affiliated Hospital of Xi'an Jiaotong University, Xi'an, China. Written informed consent for participation was not required for this study in accordance with the national legislation and the institutional requirements.

## Author contributions

R-fA: study concept and design and critical revision. FF and Y-mH: data acquisition. FF, Y-mH, and L-yW: data analysis and interpretation. YZ, P-pL, and YG: manuscript preparation. All authors contributed to the article and approved the submitted version.
