# Supplementary figures and images for: Correlation analysis of vaginal microecology and different types of human papillomavirus infection: a study conducted at a hospital in northwest China
Source: Front Med (Lausanne). 2023 Jun 1;10:1138507. doi: 10.3389/fmed.2023.1138507 (PMC10267365; doi:10.3389/fmed.2023.1138507)

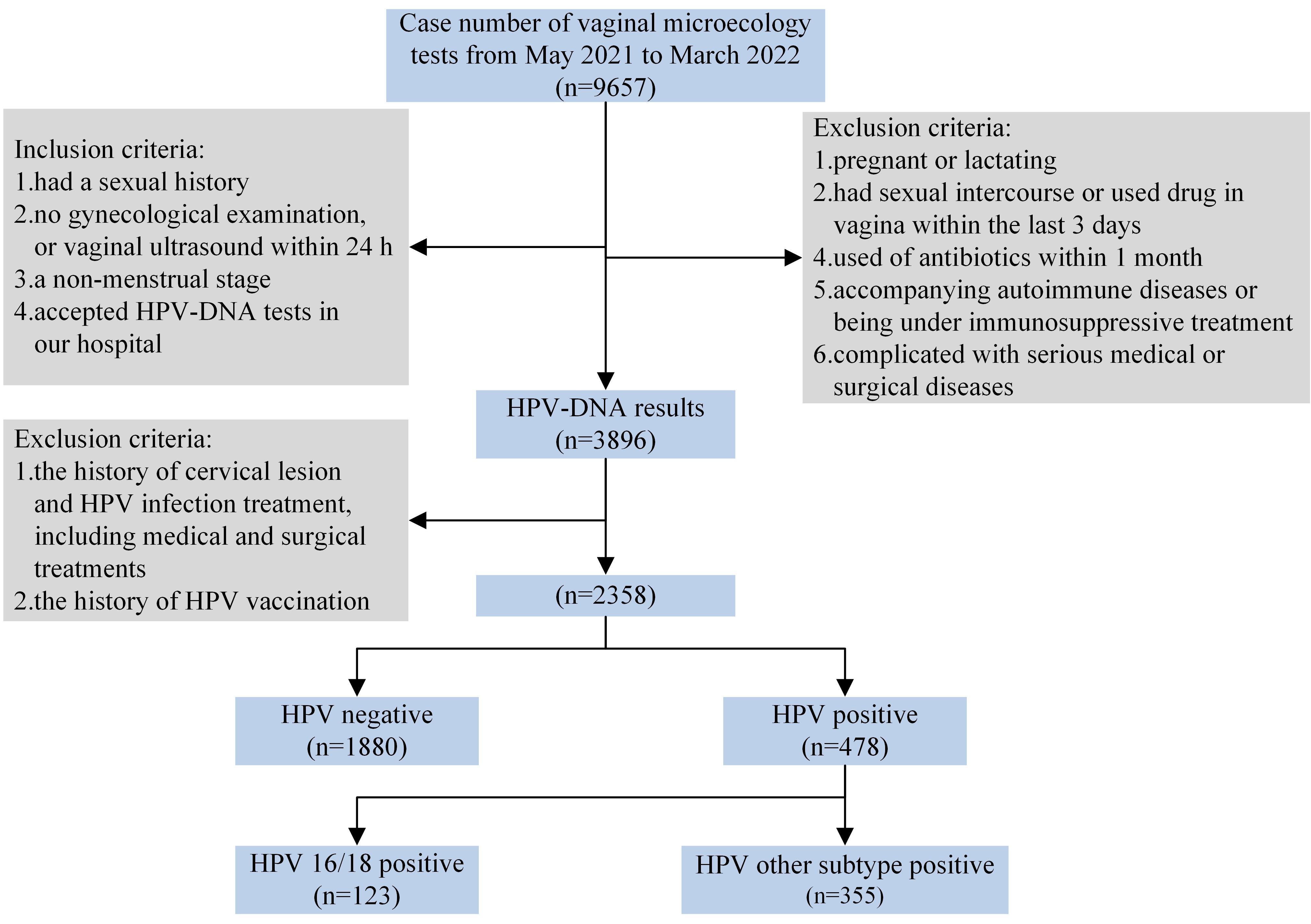

Supplement: Supplementary file 2 [file Image_1.JPEG]
